# Supplementary material for: Role of the upper airway microbiota in respiratory virus and bacterial pathobiont dynamics in the first year of life
Source: Nat Commun. 2025 Jun 4;16:5195. doi: 10.1038/s41467-025-60552-4 (PMC12137660; doi:10.1038/s41467-025-60552-4)

SUPPLEMENTARY INFORMATION

Role of the upper airway microbiota in respiratory virus and bacterial pathobiont dynamics in the first year of life

Authors: Matthew S. Kelly, MD, MPH,[1,2,3,*] Pixu Shi, PhD,[4] Sifelane C. Boiditswe, BNSc,[1] Emily Qin, BS,[4] Andrew P. Steenhoff, MBBCh,[1,5,6] Tiny Mazhani, MD,[7] Mohamed Z. Patel, MBBCh,[7] Coleen K. Cunningham, MD,[8] John F. Rawls, PhD,[3] Kathy Luinstra, HBSc,[9] Jodi Gilchrist, MSc,[9] Julia Maciejewski, MSc,[9] Jillian H. Hurst, PhD,[2] Patrick C. Seed, MD, PhD,[10] David Bulir, MD, PhD,[11] Marek Smieja, MD, PhD[9,12]

Affiliations: [1]Botswana-University of Pennsylvania Partnership, Gaborone, Botswana; [2]Division of Pediatric Infectious Diseases, Duke University, Durham, North Carolina, United States; [3]Department of Molecular Genetics and Microbiology, Duke University, Durham, North Carolina, United States; [4]Department of Biostatistics and Bioinformatics, Duke University, Durham, North Carolina, United States; [5]Global Health Center, Children's Hospital of Philadelphia, Philadelphia, Pennsylvania, United States; [6]Division of Pediatric Infectious Diseases, Children's Hospital of Philadelphia, Philadelphia, Pennsylvania, United States; [7]University of Botswana School of Medicine, Gaborone, Botswana; [8]Division of Pediatric Infectious Diseases, University of California, Irvine, Orange, California, United States; [9]Infectious Disease Research Group, Research Institute of St. Joe's Hamilton, Hamilton, Ontario, Canada; [10]Department of Pediatrics, Feinberg School of Medicine, Northwestern University, Chicago, Illinois, United States; [11]Department of Chemical Engineering, McMaster University, Hamilton, Ontario, Canada; [12]Department of Pathology and Molecular Medicine, McMaster University, Hamilton, Ontario, Canada; *corresponding author: mkelly3@uams.edu

Supplementary Table 1. Associations between respiratory virus infections and acute respiratory infection symptoms

	Sample prevalence		ARI symptoms	
	**n**	**(%)**	**n**	**(%)**
Adenovirus	65	2.7%	23	35%
Human metapneumovirus	17	0.7%	13	76%
Influenza viruses	14	0.6%	11	79%
A	13	0.5%	11	85%
B	1	<0.1%	0	0%
Parainfluenza viruses	39	1.6%	23	59%
Type 1	8	0.4%	2	25%
Type 2	4	0.2%	1	25%
Type 3	27	1.1%	20	74%
Rhinovirus/enterovirus	507	21.4%	209	41%
Respiratory syncytial virus	44	1.9%	32	73%
SARS-CoV-2	1	<0.1%	0	0%
More than one virus	48	2.0%	23	48%
No viruses	1733	73.1%	308	18%

ARI, acute respiratory infection; SARS-CoV-2, severe acute respiratory syndrome coronavirus 2

Supplementary Table 2. Associations between symptomatic and asymptomatic respiratory virus detections and the odds of acquiring bacterial pathobionts during infancy

		Intervals (n)	Colonization frequency (n, %)		Odds ratio	95% confidence interval	p
H. influenzae acquisition							
	Virus detected, ARI symptoms	205	59	29%	1.84	(1.24 - 2.73)	0.003
	Virus detected, no ARI symptoms	226	51	23%	1.13	(0.76 - 1.70)	0.54
	No viruses detected	1081	180	17%	Ref	-	-
M. catarrhalis acquisition							
	Virus detected, ARI symptoms	104	54	52%	1.72	(1.08 - 2.76)	0.02
	Virus detected, no ARI symptoms	138	55	40%	1.10	(0.72 - 1.68)	0.67
	No viruses detected	729	256	35%	Ref	-	-
S. aureus acquisition							
	Virus detected, ARI symptoms	233	14	6%	0.48	(0.26 - 0.89)	0.02
	Virus detected, no ARI symptoms	279	16	6%	0.42	(0.24 - 0.76)	0.004
	No viruses detected	1177	170	14%	Ref	-	-
S. pneumoniae acquisition							
	Virus detected, ARI symptoms	133	69	52%	2.76	(1.80 - 4.22)	<0.0001
	Virus detected, no ARI symptoms	151	51	34%	1.25	(0.82 - 1.90)	0.30
	No viruses detected	810	212	26%	Ref	-	-

Odds ratios, 95% confidence intervals, and two-sided p values were estimated from mixed effect logistic regression models adjusted for age, sex, low birth weight (<2500g), maternal HIV infection, location of residence, number of children under 5 years of age in the household, season, breastfeeding, receipt of antibiotics since the prior study visit, and 13-valent pneumococcal conjugate vaccine doses (for *S. pneumoniae* only). The p value for the odds of *S. pneumoniae* acquisition associated with respiratory virus detection with ARI symptoms, compared to no virus detection, was 2.95×10^{-6}. No adjustments were made for multiple comparisons. ARI, acute respiratory infection, Ref, reference

Supplementary Table 3. Associations between respiratory virus detections and the odds of acquiring bacterial pathobionts during infancy

		**Intervals (n)**	**Colonization frequency (n, %)**		**Odds ratio**	**95% confidence interval**	**p**
H. influenzae acquisition							
	Rhinovirus/enterovirus only	324	79	24%	1.38	(0.98 - 1.94)	0.07
	Other viruses detected	107	31	29%	1.65	(0.98 - 2.76)	0.06
	No viruses detected	1081	180	17%	Ref	-	-
M. catarrhalis acquisition							
	Rhinovirus/enterovirus only	187	79	42%	1.22	(0.84 - 1.77)	0.29
	Other viruses detected	55	30	55%	1.82	(0.96 - 3.45)	0.06
	No viruses detected	729	256	35%	Ref	-	-
S. aureus acquisition							
	Rhinovirus/enterovirus only	382	24	6%	0.46	(0.28 - 0.75)	0.002
	Other viruses detected	130	6	5%	0.39	(0.15 - 1.00)	0.0499
	No viruses detected	1177	170	14%	Ref	-	-
S. pneumoniae acquisition							
	Rhinovirus/enterovirus only	219	92	42%	1.87	(1.32 - 2.64)	0.0005
	Other viruses detected	65	28	43%	1.73	(0.95 - 3.13)	0.07
	No viruses detected	810	212	26%	Ref	-	-

Odds ratios, 95% confidence intervals, and two-sided p values were estimated from mixed effect logistic regression models adjusted for age, sex, low birth weight (<2500g), maternal HIV infection, location of residence, number of children under 5 years of age in the household, season, breastfeeding, receipt of antibiotics since the prior study visit, and 13-valent pneumococcal conjugate vaccine doses (for *S. pneumoniae* only). No adjustments were made for multiple comparisons. Ref, reference

Supplementary Table 4. Targets for multiplex PCR assay for the detection of bacterial pathobionts

Species	Primer and probe sequences
Haemophilus influenzae	F, 5’ d CCAATCACATCATCTGCAACTA 3’<br>R, 5’ d GCGTGGCTTGATGGATTG 3’<br>Probe, 5’ d CAL Fluor Red 610-TGGTTTTTCGCCTGACTCACTAAAAG-BHQ-2 3’
Moraxella catarrhalis	F, 5’ d GGTAAAAAGGTTGGAAAAACC 3’<br>R, 5’ d CTGACGCTAAGATTAGGATTGA 3’<br>Probe, 5’ d Quasar 670-CCAAGCTTTGGGGTGATTTATGATG-BHQ-2 3’
Staphylococcus aureus	F, 5’ d TGMATATTTTACCTAGTAAAGAAGG 3’<br>R, 5’ d ATACRTACATTCAAATCAGCTTC 3’<br>Probe, 5’ d HEX-TGTACGAAGAATTRGAAAGAACGATTGAG-BHQ-1 3’
Streptococcus pneumoniae	F, 5’ d AAGACTTCAATAAGCCTTCTACG 3’<br>R, 5’ d GAGAAGGTGAGRAGTCAAGAAG 3’<br>Probe, 5’d FAM-TCTTCAGACGAATTTTCTGTCATAGGAACA-BHQ-1 3’

Supplementary Figure 1. Comparison of upper respiratory tract microbiota types identified using k-medoids and k-means clustering. a. NMDS plot depicts microbiota types identified using k-medoids clustering on Bray-Curtis distances. Ellipses define the regions containing 80% of all samples that can be drawn from the underlying multivariate t distribution. **b.** Relative abundances of highly abundant genera in infant nasopharyngeal samples by k-medoids microbiota type. **c.** Classification of microbiota types using k-means clustering on Euclidean distances by k-medoids microbiota type. **d.** NMDS plot depicts microbiota types identified using k-means clustering on Euclidean distances. Ellipses define the regions containing 80% of all samples that can be drawn from the underlying multivariate t distribution. **e.** Relative abundances of highly abundant genera in infant nasopharyngeal samples by k-means microbiota type. The composition of the microbiota types identified by k-means clustering is similar to the composition of the types identified by k-medoids clustering. **f.** Relative abundances of microbiota types identified using k-medoids clustering on Bray-Curtis distances by k-means microbiota type. Overall, 1,943 of 2,235 (87%) samples with URT microbiota data had the same microbiota type classification using k-medoids or k-means clustering. URT microbiota types are named according to the bacterial genus or genera with the highest mean relative abundance: CD, *Corynebacterium/Dolosigranulum*; CDM, *Corynebacterium/Dolosigranulum/Moraxella*; *COR*, *Corynebacterium*; HAE, *Haemophilus*; MOR, *Moraxella*; STA, *Staphylococcus*; STR, *Streptococcus*; and OTH, "other." Source data are provided as a Source Data file. (ASV, amplicon sequence variant; NMDS, non-metric multidimensional scaling)

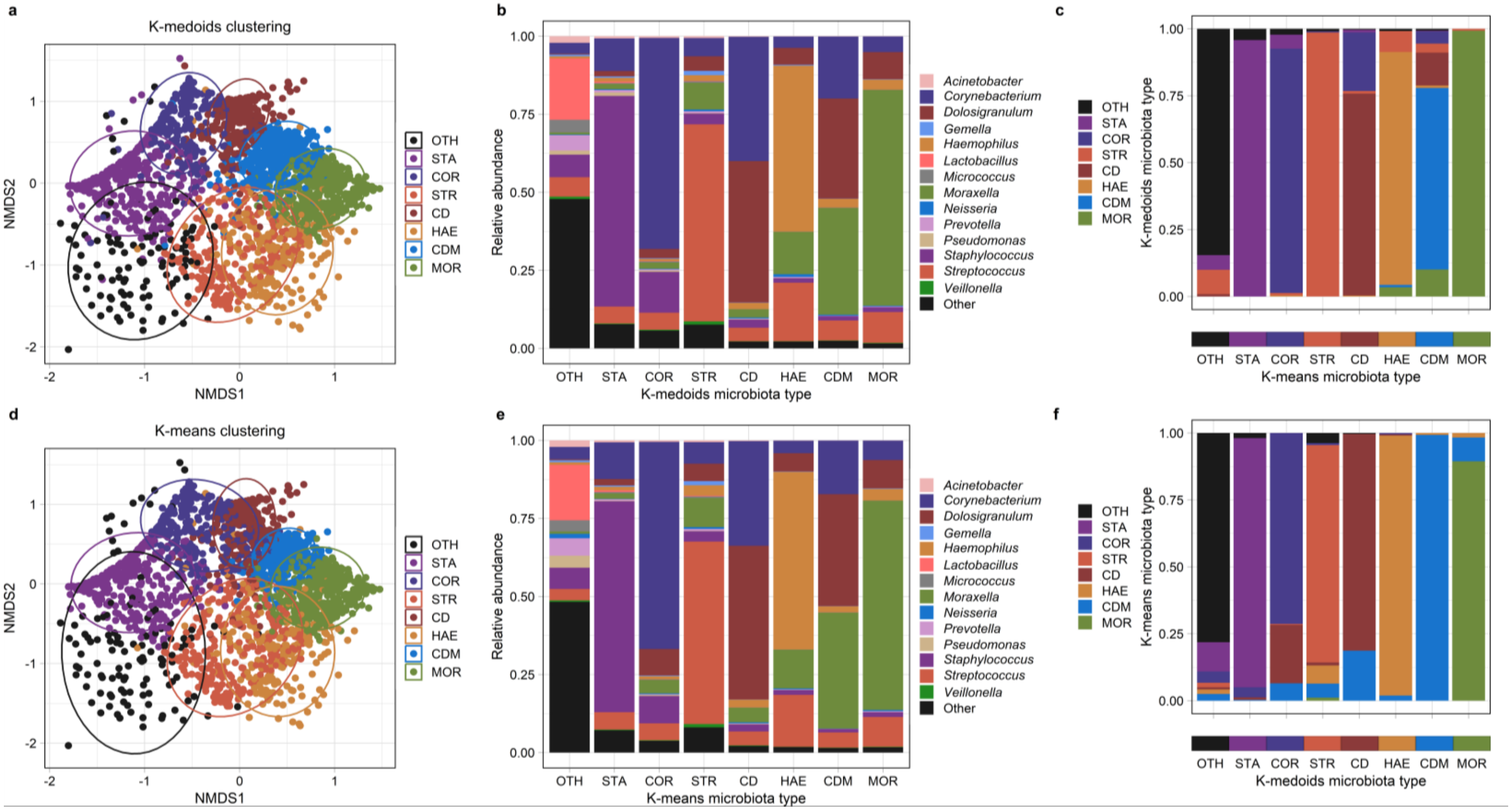

Supplementary Figure 2. Shifts in upper respiratory microbiota community states associated with the acquisition of bacterial pathobionts. Alluvial diagrams depict transitions from microbiota types associated with the acquisition of (**a.**) *H. influenzae*, (**b.**) *M. catarrhalis*, (**c.**) *S. aureus*, and (**d.**) *S. pneumoniae*. Microbiota types dominated by the genus corresponding to the bacterial pathobiont are opaque, while other microbiota types are depicted as semi-transparent. URT microbiota types are named according to the bacterial genus or genera with the highest mean relative abundance: CD, *Corynebacterium/Dolosigranulum*; CDM, *Corynebacterium/Dolosigranulum/Moraxella*; *COR*, *Corynebacterium*; HAE, *Haemophilus*; MOR, *Moraxella*; STA, *Staphylococcus*; STR, *Streptococcus*; and OTH, "other." Source data are provided as a Source Data file.

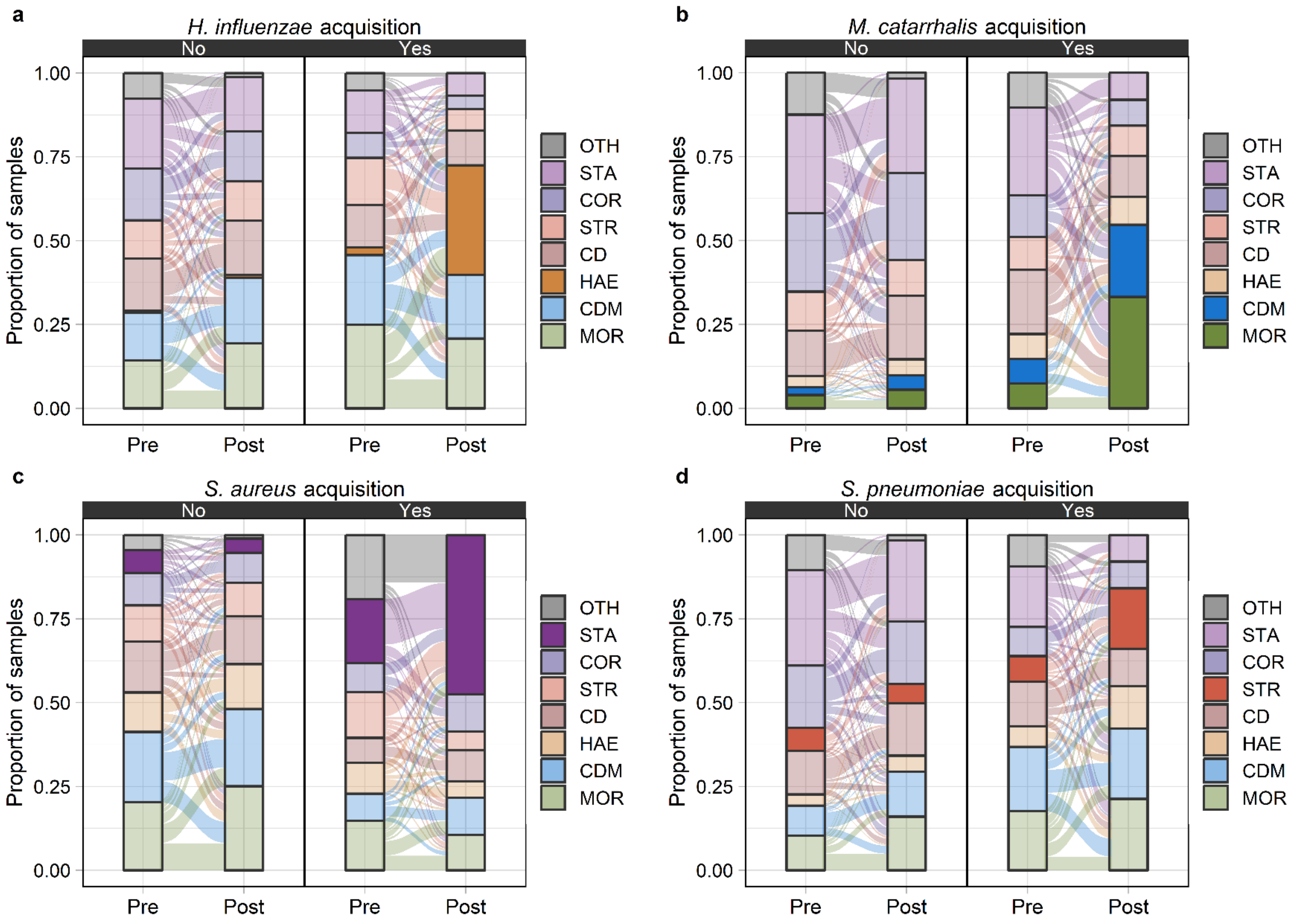

Supplementary Figure 3. Identification of ASVs containing bacterial respiratory pathobionts. a. Violin and box plots depict the relative abundances of specific ASVs within the upper respiratory microbiota by pathobiont colonization status. The line within each box indicates the median, box edges represent the 25th and 75th percentiles, and whiskers extend to 1.5 times the interquartile range. Based on BLAST searches, higher relative abundances of several ASVs potentially containing a pathobiont were observed among children identified as being colonized with that pathobiont by PCR, suggesting that these ASVs contain that pathobiont species. Specifically, higher relative abundances of ASVs 7 and 8 were observed with *H. influenzae* colonization, higher relative abundances of ASV1 were observed with *M. catarrhalis* colonization, higher relative abundances of ASV3 were observed with *S. aureus* colonization, and higher relative abundances of ASV5 were observed with *S. pneumoniae* colonization. In contrast, lower relative abundances of ASV15 were observed in children with *S. pneumoniae* colonization, consistent with this ASV being classified as other streptococcal species using BLAST. Wilcoxon rank-sum tests were used to estimate p values for differences in ASV relative abundances by pathobiont colonization status. **b.** Scatterplots show the correlation between log-transformed pneumococcal colonization density and the relative abundances of specific ASVs within the upper respiratory microbiota. Spearman's rank correlation coefficients and associated p value are depicted. A strong positive correlation (ρ=0.83) was observed between the pneumococcal colonization density by PCR and the relative abundance of ASV5, providing further evidence that this ASV contains the pathobiont *S. pneumoniae*. A weak negative correlation (ρ=-0.22) was observed between the colonization density and the relative abundance of ASV15, consistent with this ASV not containing *S. pneumoniae*. Source data are provided as a Source Data file. (ASV, amplicon sequence variant)

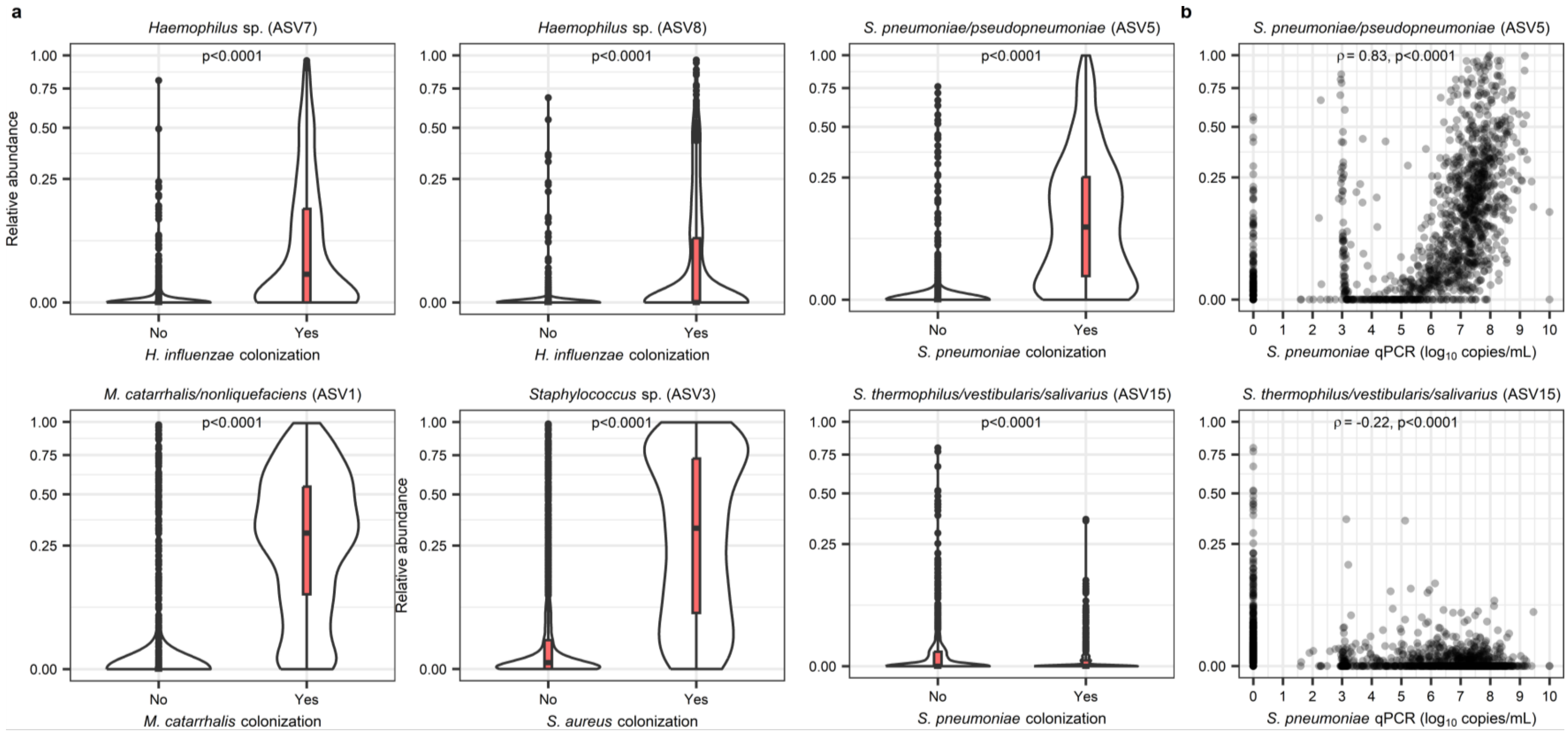

Supplement: Supplementary file 1 — Supplementary Information [file 41467_2025_60552_MOESM1_ESM.pdf]
