# Supplementary material for: Role of the upper airway microbiota in respiratory virus and bacterial pathobiont dynamics in the first year of life
Source: Nat Commun. 2025 Jun 4;16:5195. doi: 10.1038/s41467-025-60552-4 (PMC12137660; doi:10.1038/s41467-025-60552-4)
Supplement: Supplementary file 2 — Description of Additional Supplementary Files [file 41467_2025_60552_MOESM2_ESM.pdf]

Description of Additional Supplementary Files

Supplementary Data 1. STORMS Checklist
