# Supplementary material for: Role of the upper airway microbiota in respiratory virus and bacterial pathobiont dynamics in the first year of life
Source: Nat Commun. 2025 Jun 4;16:5195. doi: 10.1038/s41467-025-60552-4 (PMC12137660; doi:10.1038/s41467-025-60552-4)
Supplement: Supplementary file 4 — Reporting Summary [file 41467_2025_60552_MOESM4_ESM.pdf]

nature portfolio

Corresponding author(s): Matthew S. Kelly, MD, MPH

Last updated by author(s): 04/14/2025

Reporting Summary

Nature Portfolio wishes to improve the reproducibility of the work that we publish. This form provides structure for consistency and transparency in reporting. For further information on Nature Portfolio policies, see our Editorial Policies and the Editorial Policy Checklist.

Statistics

For all statistical analyses, confirm that the following items are present in the figure legend, table legend, main text, or Methods section.

n/a	Confirmed	
☐	☒	The exact sample size (*n*) for each experimental group/condition, given as a discrete number and unit of measurement
☐	☒	A statement on whether measurements were taken from distinct samples or whether the same sample was measured repeatedly
☐	☒	The statistical test(s) used AND whether they are one- or two-sided<br>*Only common tests should be described solely by name; describe more complex techniques in the Methods section.*
☐	☒	A description of all covariates tested
☐	☒	A description of any assumptions or corrections, such as tests of normality and adjustment for multiple comparisons
☐	☒	A full description of the statistical parameters including central tendency (e.g. means) or other basic estimates (e.g. regression coefficient) AND variation (e.g. standard deviation) or associated estimates of uncertainty (e.g. confidence intervals)
☐	☒	For null hypothesis testing, the test statistic (e.g. *F*, *t*, *r*) with confidence intervals, effect sizes, degrees of freedom and *P* value noted<br>*Give P values as exact values whenever suitable.*
☒	☐	For Bayesian analysis, information on the choice of priors and Markov chain Monte Carlo settings
☒	☐	For hierarchical and complex designs, identification of the appropriate level for tests and full reporting of outcomes
☐	☒	Estimates of effect sizes (e.g. Cohen's *d*, Pearson's *r*), indicating how they were calculated

Our web collection on statistics for biologists contains articles on many of the points above.

Software and code

Policy information about availability of computer code

Data collection	Subject characteristics were captured using REDCap.
Data analysis	All scripts used for data preprocessing and analysis are publicly available at https://github.com/mskelly7/Pathobiont_colonization.

For manuscripts utilizing custom algorithms or software that are central to the research but not yet described in published literature, software must be made available to editors and reviewers. We strongly encourage code deposition in a community repository (e.g. GitHub). See the Nature Portfolio guidelines for submitting code & software for further information.

Data

Policy information about availability of data

All manuscripts must include a data availability statement. This statement should provide the following information, where applicable:

- Accession codes, unique identifiers, or web links for publicly available datasets
- A description of any restrictions on data availability
- For clinical datasets or third party data, please ensure that the statement adheres to our policy

All data needed to reproduce the findings of this study are publicly available. The 16S rRNA gene sequencing data generated in this study have been deposited in the Sequence Read Archive under accession number PRJNA698366 [https://www.ncbi.nlm.nih.gov/sra/?term=PRJNA698366]. All metadata and processed files supporting analyses are available at https://github.com/mskelly7/Pathobiont_colonization. All files used to generate the figures in the main manuscript and Supplementary Information are included in the Source Data file.

Research involving human participants, their data, or biological material

Policy information about studies with human participants or human data. See also policy information about sex, gender (identity/presentation), and sexual orientation and race, ethnicity and racism.

Reporting on sex and gender	We reported sex for all participants - 167 (56%) infant participants were female. Sex was extracted from review of written medical records.
Reporting on race, ethnicity, or other socially relevant groupings	Race, ethnicity, and other socially defined groupings were not included in our analyses.
Population characteristics	Population characteristics included age, sex, low birth weight, maternal HIV infection, location of residence, the number of children under 5 years of age in the household, season (time-varying; dry vs. rainy), breastfeeding (time-varying), receipt of antibiotics since the prior study visit (time-varying), and PCV-13 doses.
Recruitment	The study population was drawn from four healthcare sites in and around Gaborone, Botswana, and included only mother–infant dyads meeting specific inclusion criteria (e.g., maternal age ≥18 years, singleton vaginal births, infant birth weight ≥2,000 g). As a result, the cohort may not be fully representative of the broader population of infants in Botswana, particularly those born via cesarean delivery, with low birth weight, or to adolescent mothers. Additionally, enrollment within 72 hours of delivery and the focus on selected clinical sites may have introduced bias toward families with better access to postnatal care or greater willingness to participate in research, potentially limiting the generalizability of findings.
Ethics oversight	This study was approved by the Botswana Health Research & Development Committee (HPDME 13/18/1), the Princess Marina Hospital ethics committee (PMH 5/79), and institutional review boards at the University of Pennsylvania (#822692), Duke University Health System (Pro00067434), and McMaster University (#1202). Written informed consent for use of the data and samples as described in this study was obtained from all participants or their legal guardians after a description of the study procedures in their native languages.

Note that full information on the approval of the study protocol must also be provided in the manuscript.

Field-specific reporting

Please select the one below that is the best fit for your research. If you are not sure, read the appropriate sections before making your selection.

☒ Life sciences ☐ Behavioural & social sciences ☐ Ecological, evolutionary & environmental sciences

For a reference copy of the document with all sections, see nature.com/documents/nr-reporting-summary-flat.pdf

Life sciences study design

All studies must disclose on these points even when the disclosure is negative.

Sample size	A total of 300 infants was sufficient to power our analyses for several reasons. First, the longitudinal design with repeated measures (2,409 nasopharyngeal swabs across infants) substantially increased the number of observations for modeling of colonization dynamics and temporal associations. Second, previous studies of similar design and scope have demonstrated that microbiome associations with pathobiont colonization can be reliably detected with sample sizes of <300 when paired with repeated sampling. We also observed sufficient variation in exposures of interest (e.g., viral detection, antibiotic use, environmental factors), ensuring adequate power to detect meaningful associations.
Data exclusions	For all analyses, listwise deletion was used to exclude samples or time intervals with missing data for any variable.
Replication	Biological replicates consisted of individual participants (n = 300) enrolled in a prospective birth cohort. For each participant, longitudinal nasopharyngeal swabs were collected at multiple time points; repeated measures from the same individual were not considered biological replicates. All sequencing was performed once per sample; no technical replicates were included. The analyses reflect biological variability across participants and time.
Randomization	Randomization was not applicable to this study design, as this was an observational cohort without assigned interventions. Participants were enrolled and followed prospectively, and analyses were conducted based on naturally occurring exposures and outcomes.
Blinding	Blinding was not relevant to this study design, as this was an observational cohort without treatment assignment. Microbiome data were generated using standardized laboratory protocols and processed through automated pipelines, minimizing the potential for observer bias.

Reporting for specific materials, systems and methods

We require information from authors about some types of materials, experimental systems and methods used in many studies. Here, indicate whether each material, system or method listed is relevant to your study. If you are not sure if a list item applies to your research, read the appropriate section before selecting a response.

Materials & experimental systems

n/a	Involved in the study
☒	☐ Antibodies
☒	☐ Eukaryotic cell lines
☒	☐ Palaeontology and archaeology
☒	☐ Animals and other organisms
☒	☐ Clinical data
☒	☐ Dual use research of concern
☒	☐ Plants

Methods

n/a	Involved in the study
☒	☐ ChIP-seq
☒	☐ Flow cytometry
☒	☐ MRI-based neuroimaging

Plants

Seed stocks

Report on the source of all seed stocks or other plant material used. If applicable, state the seed stock centre and catalogue number. If plant specimens were collected from the field, describe the collection location, date and sampling procedures.

Novel plant genotypes

Describe the methods by which all novel plant genotypes were produced. This includes those generated by transgenic approaches, gene editing, chemical/radiation-based mutagenesis and hybridization. For transgenic lines, describe the transformation method, the number of independent lines analyzed and the generation upon which experiments were performed. For gene-edited lines, describe the editor used, the endogenous sequence targeted for editing, the targeting guide RNA sequence (if applicable) and how the editor was applied.

Authentication

Describe any authentication procedures for each seed stock used or novel genotype generated. Describe any experiments used to assess the effect of a mutation and, where applicable, how potential secondary effects (e.g. second site T-DNA insertions, mosiacism, off-target gene editing) were examined.
